# Efficacy of Oral Sarolaner for the Treatment of Feline Otodectic Mange

**DOI:** 10.3390/pathogens10030341

**Published:** 2021-03-15

**Authors:** Diefrey Ribeiro Campos, Jéssica Karoline de Oliveira Chaves, Brena Gava Guimarães, So Yin Nak, Gabriela Pereira Salça de Almeida, Isabela Scalioni Gijsen, Juliana de Moraes Intrieri, Fabio Barbour Scott

**Affiliations:** Animal Parasitology Department, Veterinary Institute, Federal Rural University of Rio de Janeiro, BR 465, Km 07, Seropédica, RJ 23897-000, Brazil; jkochaves@ufrrj.br (J.K.d.O.C.); brenagava@ufrrj.br (B.G.G.); Soyinnak@ufrrj.br (S.Y.N.); Gabrielaalmeida@ufrrj.br (G.P.S.d.A.); gijsen@ufrrj.br (I.S.G.); julianaintrieri@ufrrj.br (J.d.M.I.); Scott.scott@ufrrj.br (F.B.S.)

**Keywords:** *Otodectes cynotis*, isoxazolines, cat

## Abstract

*Otodectes cynotis* is a mite with a cosmopolitan distribution that is the primary agent for the development of otitis externa in feline species. The aim of this study was to evaluate the efficacy of the oral administration of sarolaner for the treatment of feline otodectic mange. We used 20 adult cats of both sexes that were naturally infested with *O. cynotis*. The mite infestation scoring was performed by video-otoscopy before treatment. The cats were randomized according to the infestation score and divided into two groups (treated and control). The treated group underwent oral administration of sarolaner in a single dose of 2–4 mg/kg. The evaluations were performed by video-otoscopy to evaluate the reduction of infestation score 2, 4, 6, 24 and 48 h and 7, 14, 21 and 28 days after medication. At the end of the study, the cats were sedated to enable the recovery of live and dead mites to determine efficacy. No adverse effects or laboratory changes were observed in these cats. Sarolaner showed 100% efficacy 48 h after treatment. Based on the results, a single oral dose of sarolaner was effective in controlling otodectic mange in naturally infested cats.

## 1. Introduction

*Otodectes cynotis* (Sarcoptiformes: Psoroptidae) is a non-burrowing mite that lives on the external auditory canal epidermis. It is non-hematophagous, instead feeding on cellular debris and ear secretions. This mite can infest domestic and wild carnivores and is considered the primary agent for the development of otitis externa in cats [1]. The prevalence of this parasitic disease is higher in kittens and juvenile cats, and stray cats are also more affected than owned cats [1,2].

The clinical signs associated with otodectic mange in cats are pruritus, positive pinnal-pedal reflex, dark brown ceruminous otic exudate, head shaking and eroded ulcerated lesions in the neck and behind the ear [2]. Occasionally, some animals may have acne-like lesions caused by the mite. The development of miliary dermatitis secondary to a hypersensitive reaction to this parasite has been reported [3,4,5].

The treatment for this type of mange can be carried out locally, only in the ear canal, or systemically [6,7,8,9]. However, the biggest problem of performing just local treatment, is that in some cases, the mites that are spread over the animal's body surface can return to colonize the ear canal again [6,7]. Meanwhile, the systemic treatment has satisfactory efficacy for the control of mites in both regions [8,9].

Among the available isoxazolines, fluralaner has already been shown to be effective for the control of otodectic mange when administered orally alone [10] and associated with moxidectin transdermally [11]. Afoxolaner has also been found to be effective for the control of *O. cynotis* in cats when administered orally [12].

Efficacy has been reported of sarolaner in the treatment of feline otodectic mange when administered topically in association with selamectin [13]. However, this formulation is restricted to Europe and North America. The oral formulation of sarolaner is available globally and has proven to be effective against canine otodectic mange [14]. Furthermore, it has already been used successfully for the treatment *Lynxacarus radovskyi* [15] and *Demodex* spp. [16] in cats. There are no published studies demonstrating the isolated efficacy of orally administered sarolaner in cats for the control of *O. cynotis*. Therefore, the goal of this study was to evaluate the efficacy of sarolaner administered orally at doses of 2–4 mg/kg in cats naturally infested with *O. cynotis.*

## 2. Materials and Methods 

This study was approved by use committee of the Universidade Federal Rural do Rio de Janeiro under protocol number 2867020720 and was conducted according to the American Association of Feline Practitioners and International Society of Feline Medicine’s Feline-Friendly Handling Guidelines [17].

To evaluate the efficacy of oral sarolaner, a clinical, randomized, blinded, longitudinal and negative control study was conducted. Twenty Brazilian mixed breed (short hair) cats (12 males and 8 females), between 1 and 6 years old and weighing between 2000 and 5350 kg, were and randomly allocated to two study groups of 10 cats each. All animals belonged to the experimental cattery, had mild clinical signs of otitis externa and were naturally infested with the mite *O. cynotis*. The animals were kept in individual cages, with a floor area of about 2.5 m^2^, with visibility to other animals and with environmental enrichment.

To be included in the study, only cats that had clinical signs compatible with otitis externa and that were not carriers of chronic diseases were chosen. Laboratory analyses were performed to ensure the animals' health status, including: blood count and levels of alanine aminotransferase (ALT), aspartate aminotransferase (AST), gamma glutamyl transferase (GGT), alkaline phosphatase (ALP), albumin, urea and creatinine. These tests were performed two days before treatment (D-2), and after treatment on D+2 and D+28.

The diagnosis of otoacariasis was confirmed by bilateral video-otoscopy to assess the presence of mites. The examination was performed on days-5 and -2 with a UB-CAM Pro™ videoscope. The animals were categorized according to the number of mites present in the ear canal as observed in the video-otoscopy as described by Taenzler et al. [10], where: 0—the presence of mites was not observed; 1—fewer than five mites; 2—presence of five to ten mites; 3—more than 10 mites.

The animals were medicated with sarolaner (Simparic™—Zoetis, Brazil) following the weight recommendations for dogs. The dosages of the animals are shown in Table 1.

The presence of live mites was evaluated using video-otoscopy, with post-treatment intervals of 2, 4, 12, 24 and 48 h and then on D+7, D+14, D+21 and D+28.

After performing the otoscopic exam on D+28, the cats were sedated with a combination of xylazine (0.5 mL/kg), methadone (0.5 mg/kg) and midazolam (0.3 mL/kg) administered intramuscularly. Then, both ears of each cat were flushed to determine the number of live mites following the methods adapted by Pan et al. [18] and Taenzler et al. [10]. The external auditory canal was flushed with a 5% aqueous solution of sodium docusate, and the recovered solution was passed through filter paper to recover the mites, which were examined under a stereomicroscope (120× magnification) to count live and dead mites. The otoscopic and microscopic evaluations were performed for blinded evaluators, and, at the end of the study, all cats of the control group were treated with a topical formulation registered in Brazil for the treatment of feline otodectic mange.

The acaricidal efficacy was determined according to the scores found in the otoscopic evaluations and from counting the mites after flushing the ear canal. The percentage of efficacy against *O. cynotis* was calculated using the adapted formula from Abbott et al. [19] described below:

$$\mathrm{Efficacy}\;\left(\%\right)=\frac{\left(a\;–\;b\right)}{a}\;\times \;100$$

where: a = average of scores or average number of mites recovered in the control group, and b = average of scores or average number of mites recovered in the treated group.

For statistical analysis, the data were tabulated and the mean scores of the control and treated groups were compared using the Mann–Whitney test. To compare the average number of mites recovered in these two groups after flushing the ear canal, a *T*-test was performed followed by another *T*-test. All analyses were performed with significance set at 5% (*p* < 0.05), and all the calculations were performed using the Bioestat 5.3 statistical program.

## 3. Results

No adverse effects were observed in the animals after administration of sarolaner. In addition, no changes in the hematology or serum biochemistry of cats were detected at 48 h and 28 days after treatment (Table 2).

The average scores of the animals for the mite *O. cynotis* in the pre-treatment video-otoscopy evaluations were 2.6 and 2.7 for the control and treated groups, respectively.

The average reductions in scores in cats in the treated group after treatment were 2.6, 2.5, 2.2 and 1.2 for evaluations after two, four, six and 24 h, respectively. The scores were equal to zero from 48 h after the administration of sarolaner and remained unchanged until the evaluation 28 days after treatment. The values of acaricidal efficacy based on the scores determined by video-otoscopy are shown in Table 3.

On experimental day D+28 after otoscopy, otological lavage was performed to count the number of live mites in all cats. In the control group, the average number of live mites recovered was 20.4, while in the treated group it was zero mites. The effectiveness after flushing the external auditory canal of cats was 100.0%, as shown in Table 4.

## 4. Discussion

Although sarolaner is only indicated for the treatment of dogs, this formulation is used worldwide, and although the topical formulation of sarolaner + selamectin has already demonstrated effectiveness against the mite *O. cynotis* in cats [13] and other ectoparasites [20,21,22], its sale is restricted to North America and Europe. This association used a lower dose of sarolaner (1–2 mg/kg). However, the efficacy of sarolaner at this dose cannot be proven, since selamectin is able to eliminate 99% of mites when used topically on cats at a dose of 6 mg/kg [23].

However, although there is no indication in the package leaflet for the oral use of sarolaner in cats, it has already been used successfully for the treatment of feline demodicosis [16] and lynxacariosis [15], and the authors did not report any clinical or laboratory changes in the animals, as was the case in this study (Table S1)

According to McTier et al. [24], sarolaner reached high concentrations 24 h after oral administration in dogs. Therefore, the pharmacokinetics are probably similar in cats, since in this study sarolaner showed 100% effectiveness in controlling *O. cynotis* after 48 h.

According to Kennis [8] and Moriello [9], treatments administered systemically may have better results in controlling mites that cause otitis externa in cats, since they are able to completely eliminate parasites on the animal's body. Our results corroborate that finding: sarolaner administered orally was more effective than that reported by Becskei et al. [13] using a formulation of sarolaner and selamectin, administered topically, which obtained approximately 99% effectiveness 30 days after treatment.

Other isoxazolines also have been used with similar efficacy to that found in this study. Fluralaner administered orally was 100% effective against the mite *O. cynotis* 28 days after treatment [10]. Afoxolaner administered orally at a dose of 2.5 mg/kg was 100% effective for the treatment of otodectic mange seven days after treatment [12].

The use of ectoparasiticides orally in cats may encounter resistance from their owners, considering the difficulty of administering tablets to feline species. However, this type of treatment allows for reducing the risk of accidental ingestion of large amounts of ectoparasiticides and consequent intoxication caused by allogrooming in houses with several animals. Associated with this, systemic treatment (orally) both reduces the incidence of environmental contamination and exposure of pet owners to residues, considering that damage to human health is not yet known [25,26].

To the best of our knowledge, this is the first study to evaluate the efficacy of orally administered sarolaner for the treatment of feline otodectic mange. The treatment produced a satisfactory acaricidal result, so it can be considered a good option for the resolution of *O. cynotis* infestations in cats.

## 5. Conclusions

On the basis of the results demonstrated in this study, it is possible to conclude that sarolaner, when administered orally, is effective for the treatment of feline otodectic mange.

## Figures and Tables

**Table 1:** Identification, sex, age, weight, and dose of sarolaner per tablet administered to the cats in the study.

Animals	Sex	Age (years)	Weight (kg)	Administered Tablet (mg)	Dose (mg/kg)
**1**	Female	4	5.100	20	3.9
**2**	Male	6	5.150	20	3.9
**3**	Female	3	3.600	10	2.8
**4**	Male	2	4.200	10	2.4
**5**	Male	3	3.600	10	2.8
**6**	Female	6	3.100	10	3.2
**7**	Male	4	5.200	20	3.8
**8**	Female	4	3.550	10	2.8
**9**	Male	3	3.750	10	2.7
**10**	Male	1	2.900	10	3.4
**11**	Male	1	5.350	- - -	- - -
**12**	Male	3	5.050	- - -	- - -
**13**	Male	4	4.200	- - -	- - -
**14**	Female	4	3.150	- - -	- - -
**15**	Female	6	3.850	- - -	- - -
**16**	Female	3	3.750	- - -	- - -
**17**	Male	3	4.100	- - -	- - -
**18**	Male	4	3.900	- - -	- - -
**19**	Female	2	2.000	- - -	- - -
**20**	Male	2	3.750	- - -	- - -

**Table 2:** Hematological and serum biochemical parameters of the control group and group treated with a single oral dose of sarolaner (2–4 mg/kg).

Parameters	Control Mean (Standard Deviation SD)			Treated Mean (SD)			Reference Interval
	D-2	D2	D30	D-2	D2	D30	
Blood Count							
RBC (×10^6^/µL)	8.2 ^a^ (0.6)	8.4 ^c^ (0.5)	8.4 ^e^ (0.8)	8.6 ^a^ (0.7)	8.5 (0.5)	8.9 ^e^ (0.6)	5.92–9.93
Haemoglobin (g/dL)	12.4 ^a^ (0.9)	12.8 ^c^ (1.7)	12.6 ^e^ (1.1)	12.2 ^a^ (0.4)	12.6 ^c^ (1.7)	12.4 ^e^ (1.3)	9.3–15.9
Haematocrit (%)	35.5 ^a^ (2.6)	37.2 ^c^ (2.9)	35.8 ^e^ (4.2)	38.2 ^a^ (4.1)	37.8 ^c^ (3.9)	38.9 ^e^ (3.7)	29–48
MCV (fL)	44.9 ^a^ (4.8)	43.8 ^c^ (3.2)	42.5 ^e^ (4.5)	42.3 ^a^ (3.7)	41.6 ^c^ (2.9)	42.6 ^e^ (3.3)	37–61
MCHC (g/dL)	32.1 ^a^ (1.2)	31.3 ^c^ (1.0)	31.9 ^e^ (0.7)	31.4 ^a^ (0.9)	32.5 ^c^ (0.7)	32.9 ^e^ (1.1)	30–38
WBC (/µL)	14,942 ^a^ (6505)	11,907 ^c^ (3744)	12,050 ^e^ (4246)	13,259 ^a^ (4287)	15,871 ^c^ (5369)	12,189 ^e^ (4548)	3.5–16
Neutrophils (/µL)	6436 ^a^ (4004)	7611 ^c^ (2429)	6353 ^e^ (3100)	6127 ^a^ (2934)	5297 ^c^ (3843)	7402 ^e^ (4161)	2500–8900
Lymphocytes (/µL)	2632 ^a^ (1075)	4300 ^c^ (2318)	4921 (3403)	2459 ^a^ (1899)	4311 ^c^ (3319)	3467 ^e^ (2435)	1200–8500
Eosinophils (/µL)	632 ^a^ (93)	877 ^c^ (842)	920 ^e^ (328)	990 ^a^ (97)	833 ^c^ (46)	932 (88)	0–1000
Monocytes (/µL)	160 ^a^ (87)	261 ^c^ (115)	321 ^e^ (97)	132 ^a^ (43)	321 (154)	295 (228)	0–600
Platelets(×10^3^/µL)	276.0 ^a^ (867.9)	254.9 ^c^ (733.5)	296.1 ^e^ (854.2)	252.2 ^a^ (223.7)	242.8 ^c^ (374.8)	233.9 ^e^ (378.2)	200–500
**Serum biochemistry**							
ALT (U/L)	86.2 ^a^ (7.3)	74.6 ^c^ (18.6)	64.1 ^e^ (12.3)	94.4 ^a^ (19.7)	85.9 ^c^ (21.2)	58.7 ^e^ (16.3)	10–100
AST (U/L)	44.7 ^a^ (13.7)	58.8 ^c^ (15.1)	40.2 ^e^ (12.9)	37.8 ^a^ (10.1)	66.1 ^c^ (12.3)	45.2 ^e^ (10.1)	10–100
AP (U/L)	31.3 ^a^ (6.8)	35.7 ^c^ (10.4)	56.7 ^e^ (12.1)	37.4 ^a^ (11.9)	52.4 ^c^ (13.9)	48.9 ^e^ (15.1)	6–102
GGT (U/L)	1.7 ^a^ (0.2)	2.1 ^c^ (0.6).	1.2 ^e^ (0.3)	1.4 ^a^ (0.5)	1.9 ^c^ (1.1)	2.1 ^e^ (0.5)	1–10
Albumin (g/dL)	2.8 ^a^ (0.5)	2.7 ^c^ (0.6)	2.6 ^e^ (0.6)	2.7 ^a^ (0.4)	2.5 ^c^ (0.5)	2.8 ^e^ (0.5)	2.5–3.9
Urea (mg/dL)	35.0 ^a^ (12.7)	21.0 ^c^ (11.2)	33.4 ^e^ (4.8)	31.1 ^a^ (6.8)	29.9 ^c^ (8.6)	31.1 ^e^ (8.1)	14–36
Creatinine (mg/dL)	1.2 ^a^ (0.2)	1.3 ^c^ (0.3)	1.1 ^e^ (0.2)	1.1 ^a^ (0.4)	1.3 ^c^ (0.2)	1.1 ^e^ (0.3)	0.6–1.8

Mann–Whitney test (*p* < 0.05). Different letters indicate statistically significant differences (^a^ D-2; ^c^ D+2; ^e^ D+30). RBC = red blood cells, MVC = mean corpuscular volume, CHCM = mean corpuscular hemoglobin concentration, WBC = white blood cells, ALT = alanine aminotransferase, AST = aspartate transaminase, AP = alkaline phosphatase, GGT = gamma glutamyl transferase.

**Table 3:** Efficacy of sarolaner, administered orally, against the mite *Otodectes cynotis* in naturally infested cats.

Experimental Day	Control Group	Treated Group	Efficacy	*p*-Value
	Mean ± Standard Deviation	Mean ± Standard Deviation		
**-5**	2.6 ± 0.5	2.7 ± 0.5	- - -	0.6477
**D-2**	2.6 ± 0.5	2.7 ± 0.5	- - -	0.6477
**D0+2 h**	2.7 ± 0.5	2.6 ± 0.5	3.7	0.6477
**D0+4 h**	2.7 ± 0.5	2.5 ± 0.5	7.4	0.3736
**D0+12 h**	2.6 ± 0.5	2.2 ± 0.8	15.4	0.2402
**D+1**	2.6 ± 0.5	1.2 ± 0.8	53.8	0.0015
**D+2**	2.6 ± 0.5	0.0 ± 0.0	100	0.0002
**D+7**	2.6 ± 0.5	0.0 ± 0.0	100	0.0002
**D+14**	2.7 ± 0.5	0.0 ± 0.0	100	0.0002
**D+21**	2.7 ± 0.5	0.0 ± 0.0	100	0.0002
**D+28**	2.8 ± 0.4	0.0 ± 0.0	100	0.0002

**Table 4:** Number of live mites (*Otodectes cynotis*) recovered after otological flushing and acaricidal efficacy of orally administered sarolaner.

Group		Right Ear	Left Ear	Total
Control	Mean ± SD	11.2 ± 6.9	9.2 ± 4.6	20.4 ± 10.7
Treated	Mean ± SD	0.0 ± 0.0	0.0 ± 0.0	0
	Efficacy	- - -	- - -	100%
	*p*-value	- - -	- - -	<0.0001

## Data Availability

The data presented in this study are available in the main text or in Supplementary Materials.

## Supplementary Materials

The following are available online at https://www.mdpi.com/2076-0817/10/3/341/s1, Table S1: Individual results of the efficacy of sarolaner administered orally against the mite *Otodectes cynotis* in naturally infested cats. Table S2: Number of live mites (*Otodectes cynotis*) recovered after ear flush and the acaricidal efficacy of orally administered sarolaner in the treated group.
