# Supplementary material for: Efficacy of Oral Sarolaner for the Treatment of Feline Otodectic Mange
Source: Pathogens. 2021 Mar 15;10(3):341. doi: 10.3390/pathogens10030341 (PMC8001191; doi:10.3390/pathogens10030341)
Supplement: Supplementary file 1 [file pathogens-10-00341-s001.pdf]

Supplementary Table S1. Individual results of the efficacy of sarolaner, administered orally, against the mite *Otodectes cynotis* in naturally infested cats.

Group	Animal	Experimental Day										
		D-5	D-2	D0 +2h	D0+4h	D0+12h	D+1	D+2	D+7	D+14	D+21	D+28
Control	**1**	3	3	3	3	3	3	3	3	3	3	3
	**2**	2	2	2	2	2	2	2	2	2	2	2
	**3**	3	3	3	3	2	3	3	3	3	3	3
	**4**	2	2	2	2	2	2	2	3	3	3	3
	**5**	3	3	3	3	3	3	3	3	2	2	3
	**6**	3	3	3	3	3	3	3	3	3	3	3
	**7**	2	2	2	2	2	2	2	2	3	3	3
	**8**	3	3	3	3	3	3	3	3	3	3	3
	**9**	2	2	3	3	3	2	2	2	2	2	2
	**10**	3	3	3	3	3	3	3	2	3	3	3
	**Mean**	**2.6**	**2.6**	**2.7**	**2.7**	**2.6**	**2.6**	**2.6**	**2.6**	**2.7**	**2.7**	**2.8**
	**Standard Deviation**	**0.5**	**0.5**	**0.5**	**0.5**	**0.5**	**0.5**	**0.5**	**0.5**	**0.5**	**0.5**	**0.4**
Treated	**11**	3	3	3	3	3	1	0	0	0	0	0
	**12**	2	2	2	2	1	1	0	0	0	0	0
	**13**	3	3	3	3	3	1	0	0	0	0	0
	**14**	3	3	3	2	2	2	0	0	0	0	0
	**15**	2	2	2	2	2	2	0	0	0	0	0
	**16**	3	3	3	3	3	2	0	0	0	0	0
	**17**	3	3	2	2	1	0	0	0	0	0	0
	**18**	3	3	3	3	2	1	0	0	0	0	0
	**19**	3	3	3	3	3	2	0	0	0	0	0
	**20**	2	2	2	2	2	0	0	0	0	0	0
	**Mean**	**2.7**	**2.7**	**2.6**	**2.5**	**2.2**	**1.2**	**0**	**0**	**0**	**0**	**0**
	**Standard Deviation**	**0.5**	**0.5**	**0.5**	**0.5**	**0.8**	**0.8**	**0.0**	**0.0**	**0.0**	**0.0**	**0.0**
	**Efficacy**	- - -	- - -	**3.7**	**7.4**	**15.4**	**53.8**	**100.0**	**100.0**	**100.0**	**100.0**	**100.0**
	**P- value**	0.6477	0.6477	0.6477	0.3736	0.2402	0.0015	0.0002	0.0002	0.0002	0.0002	0.0002

Supplementary Table S2: Number of live mites (*Otodectes cynotis*) recovered after each cat's ear flush in the study and the acaricidal efficacy of orally administered sarolaner in the treated group.

Group	Animal	Right ear	Left ear	Total
Control	**1**	16	13	29
	**2**	4	3	7
	**3**	12	11	23
	**4**	7	2	9
	**5**	23	11	34
	**6**	18	12	30
	**7**	4	5	9
	**8**	12	13	25
	**9**	2	7	9
	**10**	14	15	29
	**Mean**	**11,2**	**9,2**	**20,4**
	**Standard Deviation**	**6.9**	**4.6**	**10.7**
Treated	**11**	0	0	0
	**12**	0	0	0
	**13**	0	0	0
	**14**	0	0	0
	**15**	0	0	0
	**16**	0	0	0
	**17**	0	0	0
	**18**	0	0	0
	**19**	0	0	0
	**20**	0	0	0
	**Mean**	**0**	**0**	**0**
	**Standard Deviation**	**0,0**	**0,0**	**0,0**
	**Efficacy**	- - -	- - -	100
	p- value	- - -	- - -	<0.0001
